# Genomic insights into *Plasmodium vivax* population structure and diversity in central Africa

**DOI:** 10.1186/s12936-024-04852-y

**Published:** 2024-01-18

**Authors:** Valerie Gartner, Benjamin D. Redelings, Claudia Gaither, Jonathan B. Parr, Albert Kalonji, Fernandine Phanzu, Nicholas F. Brazeau, Jonathan J. Juliano, Gregory A. Wray

**Affiliations:** 1 Biology Department, Duke University, Durham, NC 27708 USA; 2 University Program in Genetics and Genomics, Duke University, Durham, NC 27708 USA; 3 Department of Ecology and Evolutionary Biology, University of Kansas, Lawrence, KS 66045 USA; 4 Ronin Institute, Durham, NC 27705 USA; 5 University of North Carolina, Chapel Hill, NC 27599 USA; 6 SANRU Asbl, 149 A/B, Boulevard du 30 Juin, Kinshasa, Gombe, Democratic Republic of Congo; 7 Duke University Medical Center, Durham, NC 27708 USA

**Keywords:** Malaria, *Plasmodium vivax*, Genome, Duffy negative, Sub-Saharan Africa, Central Africa

## Abstract

**Background:**

Though *Plasmodium vivax* is the second most common malaria species to infect humans, it has not traditionally been considered a major human health concern in central Africa given the high prevalence of the human Duffy-negative phenotype that is believed to prevent infection. Increasing reports of asymptomatic and symptomatic infections in Duffy-negative individuals throughout Africa raise the possibility that *P. vivax* is evolving to evade host resistance, but there are few parasite samples with genomic data available from this part of the world.

**Methods:**

Whole genome sequencing of one new *P. vivax* isolate from the Democratic Republic of the Congo (DRC) was performed and used in population genomics analyses to assess how this central African isolate fits into the global context of this species.

**Results:**

*Plasmodium vivax* from DRC is similar to other African populations and is not closely related to the non-human primate parasite *P. vivax*-like. Evidence is found for a duplication of the gene PvDBP and a single copy of PvDBP2.

**Conclusion:**

These results suggest an endemic *P. vivax* population is present in central Africa. Intentional sampling of *P. vivax* across Africa would further contextualize this sample within African *P. vivax* diversity and shed light on the mechanisms of infection in Duffy negative individuals. These results are limited by the uncertainty of how representative this single sample is of the larger population of *P. vivax* in central Africa.

**Supplementary Information:**

The online version contains supplementary material available at 10.1186/s12936-024-04852-y.

## Background

The widespread fixation of the Duffy-negative phenotype in the human population in sub-Saharan Africa, which provides protection from *Plasmodium vivax*, is one of the most remarkable cases of natural selection documented in human populations [1–3]. The Duffy-negative phenotype occurs in humans with two copies of a silencing mutation in the promoter region of the Duffy Antigen Receptor for Chemokines (DARC) gene, resulting in the absence of receptor expression exclusively in erythrocytes necessary for the progression of the *P. vivax* life cycle [4, 5]. Despite this, there are an increasing number of reports of asymptomatic and symptomatic *P. vivax* infections in people with the Duffy-negative mutation suggesting that *P. vivax* persists in central Africa at low levels in people with the Duffy-negative resistance allele [6–10].

An alternate explanation for the persistence of *P. vivax* in central Africa comes from the recent discovery by [11] of a closely related parasite species that infects non-human primates, *P. vivax*-like, in Western Africa [11–14]. Though there is only one confirmed report of *P. vivax*-like infecting a Duffy-positive Caucasian traveller [11], a study using *P. vivax*-like recombinant binding proteins did not reveal species-specific barriers to erythrocyte invasion of human, gorilla, or chimpanzee red blood cells, suggesting *P. vivax*-like likely is able to infect humans [13].

A third possible explanation for the presence of *P. vivax* in central Africa despite human resistance alleles might be that *P. vivax* is adapting to overcome the Duffy negative resistance allele, which would be a serious concern for malaria elimination efforts in central Africa. Genomics can potentially aid in understanding the source of these infections in central Africa, however none of the seventy-seven publicly available African *P. vivax* genomes are from regions with high levels of Duffy negativity except for three samples from Uganda. Importantly, these Ugandan samples were collected from people of unknown Duffy status after returning to the UK [15, 16].

In this study, whole genome sequencing of a new *P. vivax* isolate from the Eastern region of Democratic Republic of the Congo (DRC) is performed to assess how *P. vivax* from Central Africa fits into the global context of this pathogen. Though the original study design excludes the possibility of genotyping the human host of this *P. vivax* sample, the patient had no known travel history and resides in a region where the Duffy-negative phenotype frequency is at or above 80% [17], thus this patient has a high chance of having the Duffy-negative phenotype. The presence of a *P. vivax* population in central Africa that is not closely related to the ape-infection *P. vivax*-like species is confirmed. Further, this sample was investigated for duplications of the Duffy binding ligand genes PvDBP and PvDPB2 (also referred to as EBP and EBP2) which might potentially enable *P. vivax* to evade host immunity. Though copy number variation of these genes is not conclusively linked to *P. vivax* infection of Duffy-negative individuals [18, 19], both genes contain the Duffy Binding Protein II domain, one of the foremost vaccine target candidates [20]. Evidence is found for a duplication of the gene PvDBP in the DRC *P. vivax* sample and a single copy of PvDBP2.

## Methods

### Genome sequencing of *Plasmodium vivax* sample from the Democratic Republic of Congo

One *P. vivax* sample was collected from Idjwi, DRC [21]. The patient was an 11-year-old with reported fever, diarrhoea, and headache who tested positive for *P. vivax* via 18s qPCR assay as previously described [6] at 957 parasites per µL. The patient tested negative for *P. falciparum* via rapid HRP2 test and real time PCR. Due to the original study design, the patient’s Duffy genotype was not assessed. Travel history was not taken.

DNA from three 6 mm punches from a dried blood spot were extracted using Chelex-Tween as previously described [22] *P. vivax* infection was confirmed using a Taqman real time PCR assay [6]. *Plasmodium* DNA was enriched from human DNA using a custom Twist hybrid capture array and in-house pipeline (Twist Biosciences, San Francisco, CA, USA). The array was designed by single tiling of the PvP01 genome with baits complementary to human removed. Capture and library preparation were completed per manufacturer’s instructions. Sequencing was completed on a NovaSeq 6000 at the University of North Carolina High Throughput Sequencing Facility.

### Genomic data processing

1408 FASTQ files for *P. vivax* with metadata about the geographic location from which they were sampled were downloaded from the Sequence Read Archive [23]. BAM files were created using bwa mem [24] to align short reads to the PvP01 reference genome [25]. Picard MarkDuplicates version 2.18.15 [26] was used to remove optical duplicates, and variants underwent hard filtering using the Genome Analysis Toolkit (GATK) HaplotypeCaller version 3.8.1, followed by joint calling [27]. To avoid confounding analyses with *P. vivax* samples made up of more than one haplotype background (i.e. a multiplicity of infection greater than one), samples were filtered out based on haplotype number estimates generated by Octopus [28]. Sample accessions and location metadata of this final sample set of 696 *P. vivax* samples, one being the new DRC genome sequenced in this paper, are available in Additional file 2: Table S5 and via the project GitHub repository: https://github.com/vlrieg/DRC_vivax/blob/main/sample_info/metadata_table.csv.

### Population genetics analysis

Analyses were performed only on assembled chromosomes as defined by the PvP01 reference genome [25]. Hyper-variable regions determined in [29] were converted to PvP01 coordinates using the alignment-smc tool in Bali-Phy with the translate-mask option [30], then removed from the data set using BEDTools version 2.25.0 [31] and VCFtools version 0.1.15 [32]. The data were reduced to biallelic Single Nucleotide Polymorphisms (SNPs) only, and LD pruning was performed using PLINK to obtain unlinked singletons and variants from the data set as previously described [16, 33] resulting in 94,083 SNPs. Principal Component Analysis was performed using Plink version 1.9 [34] and plotted in R using ggplot2 [35]. Admixture analysis was performed using Admixture software version 1.3.0 [36] via admixturePipeline version 2.0.2 [37]. The resulting Q matrices were visualized using Pong version 1.5 [38]. Cross validation error supports K = 14 populations. F4 statistics were calculated on 467,205 biallelic SNPs that had no more than 5% missing sites from 696 *P. vivax* and 56 *P. vivax*-like samples using Admixtools2 version 2.0.0 [39]. Summary statistics were computed on 696 *P. vivax* samples. π was computed on biallelic SNPs using Pixy version 1.2.4.beta1 [40].

### Phylogenetics analysis

Phylogenies of global and African subsamples of *P. vivax* were made using IQtree version 1.6.12 for Linux 64-bit using both ultrafast bootstrap approximation (UFboot) and SH-like approximate likelihood ratio test (SH-aLRT) methods to assess branch support [41–43]. Trees were constructed using biallelic SNPs from PvP01-defined nuclear chromosomes except for hypervariable regions. Phylogenies were inferred using the GTR + ASC model to account for ascertainment bias. Trees were rooted using two *P. vivax*-like samples from two recent studies of this closely related species [13, 14]. Trees were visualized using FigTree version 1.4.4 (http://tree.bio.ed.ac.uk/software/figtree/) and modified with Adobe Illustrator.

### Duffy binding gene copy number variation analysis

The read depths of important genes related to *P. vivax* pathogenesis were investigated by extracting the genomic regions from the BAM file (after removing optical duplicates) using Samtools version 1.3.1 [44] and visualized in IGV version 2.4.14 [45]. Genomic coverage as defined by read depth was calculated for PvDBP using bedtools version 2.25.0 [31]. Breakpoint evidence to support a duplication of PvDBP was estimated using Lumpy version 0.2.13 [46].

## Results

### *Plasmodium vivax* from DRC falls within African diversity in context of global population structure

**Fig. 1:**
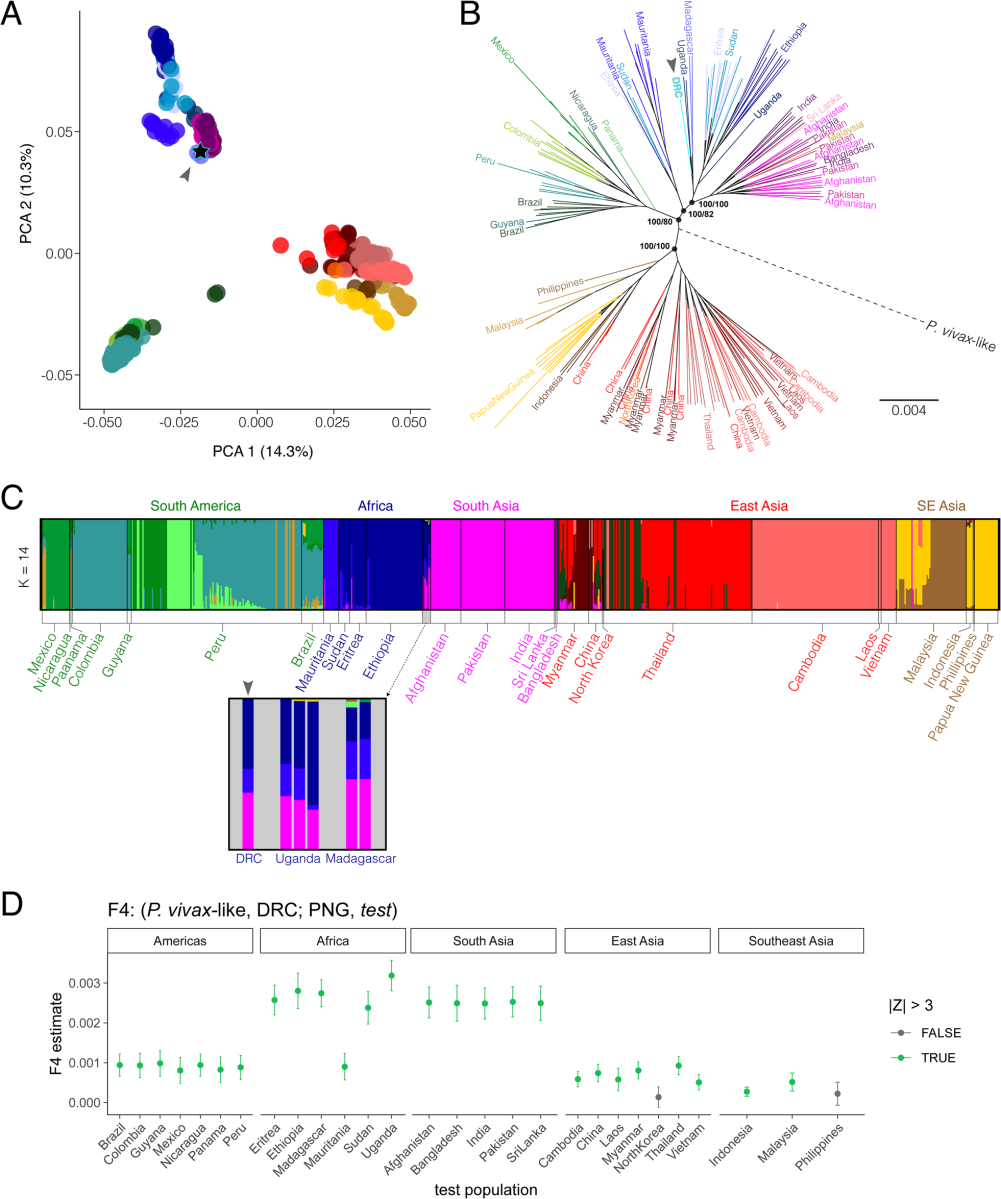
DRC *P. vivax* falls within African variation in global species context. DRC sample indicated by arrowhead in each panel. **A** Principal components analysis of global genetic diversity reveals *P. vivax* from the DRC grouped with African and South Asian populations. The first principal component explains 14.3% of variation in the data and the second principal component represents 10.3% of variation. **B** Maximum Likelihood tree shows *P. vivax* from the DRC clusters with Uganda and Madagascar. Tree constructed using whole-genome SNP data and rooted using two *P. vivax*-like sequences (red dotted line). Continent colours: Americas (green), Africa (blue), South Asia (pink), East Asia (red), Southeast Asia (brown). Tree constructed using IQtree with the GTR + ASC model to account for ascertainment bias in SNP data. Population-level SH-aLRT and UFBoot support values generated by IQTree are shown on the node in the format: SH-aLRT support (%)/ultrafast bootstrap support (%). **C** Admixture analysis of global population structure indicates *P. vivax* ancestry is geographically structured. Each vertical bar represents the proportion of genetic ancestry belonging to one individual *P. vivax* sample for each simulated population size (K). Population size K = 14 is most-supported by Cross Validation Error. **D** F4 statistics calculated using Admixtools2 using the relationship (*P. vivax*-like, DRC; Papua New Guinea (PNG), *test*). Higher F4 estimates indicate DRC has a closer relationship with the test population than it does to the PNG population. Error bars indicate ± 3 SE

To determine where this new central African sample fits within global *P. vivax* populations, principal components analysis (PCA) was first performed from 696 *P. vivax* samples using only biallelic SNPs (excluding hyper-variable regions defined by [25, 29]). The global PCA analysis in Fig. 1A shows a population structure as defined by geography is reproduced by the first two principal components, as has been reported previously [16, 33]. Three main sub-populations are formed: 1. samples from the Americas, 2. African and South Asian samples, and 3. East Asian and Southeast Asian samples. Within the cluster of African and South Asian samples, the new DRC sample is most similar to those from Uganda and Madagascar in their position alongside South Asian *P. vivax*.

To further understand how this new DRC *P. vivax* sample relates to other global populations, a maximum likelihood tree was constructed from 349,353 SNPs across the genome for no more than 10 samples per country. Figure 1B shows the DRC sample clusters within African *P. vivax* variation, and again clusters most closely with Uganda and Madagascar samples. *Plasmodium vivax*-like samples were used to root this tree, and notably, as in [33], the root for this tree is located centrally in the *P. vivax* tree and not inside African variation, which might be expected if this sample represented an ancestral source population of *P. vivax* in humans.

Though clustering of genetic variation is expected due to geographic separation, PCA and phylogenetic trees do not quantify how much genetic ancestry is shared across geographic populations. To determine the fraction of shared ancestry across subgroups, the same SNP data set used in Fig. 1A, B was assessed through Admixture analysis. *Plasmodium vivax* ancestry proportions were modelled for population sizes of 2 through 17. A population size of K = 14 was supported based on mean Cross Validation Error value. Figure 1C shows the global ancestry proportions of *P. vivax* when modelled at K = 14. Ancestry proportions for all calculated K values is shown in Additional file 1: Fig. S3. Additionally, F4 statistics [39, 47] were calculated assess the correlation in allele frequencies of the DRC sample with other *P. vivax* populations around the world. The form (*P. vivax*-like, DRC; Papua New Guinea, Y) is used here, where *P. vivax*-like is the outgroup species, shown in Fig. 1D. Higher F4 estimates indicate the DRC sample has more gene flow with the test population than it does with samples from Papua New Guinea (PNG). All test populations except for North Korea and the Philippines resulted in a significant absolute Z score (|Z| > 3). F4 estimates and related data are available in Additional file 1: Table S4.

### *Plasmodium vivax* from DRC has similar levels of population diversity as other African populations

In order to explore *P. vivax* population diversity despite having only a single sample from the DRC, the number of private alleles in each country were calculated. Private alleles are variants present in one population and in none of the others, making them unique to a population. Additional file 1: Table S1 shows the full set of summary statistics calculated for all countries. When normalizing the private allele count by dividing by the number of samples, as shown in Additional file 1: Fig. S1B, the DRC sample had a similar amount of variation as other African populations despite having a low absolute private allele count (Additional file 1: Fig. S1A). The genome-wide within-population diversity value, π, calculated for *P. vivax* from different sub-regions shown in Additional file 1: Fig. S2 indicates that when combined, all African samples have similar genome-wide diversity as populations in Asia. *P. vivax* nucleotide diversity of central African samples (DRC and Uganda) is similar to but slightly lower than that of East African (Ethiopia, Eritrea, and Sudan). Caution must be used in the interpretation of this result, however, as the single DRC sample and three Ugandan samples are unable to reflect the full scope of *P. vivax* genetic diversity in this region.

### *Plasmodium vivax* in central Africa is distinct from *Plasmodium vivax*-like

**Fig. 2:**
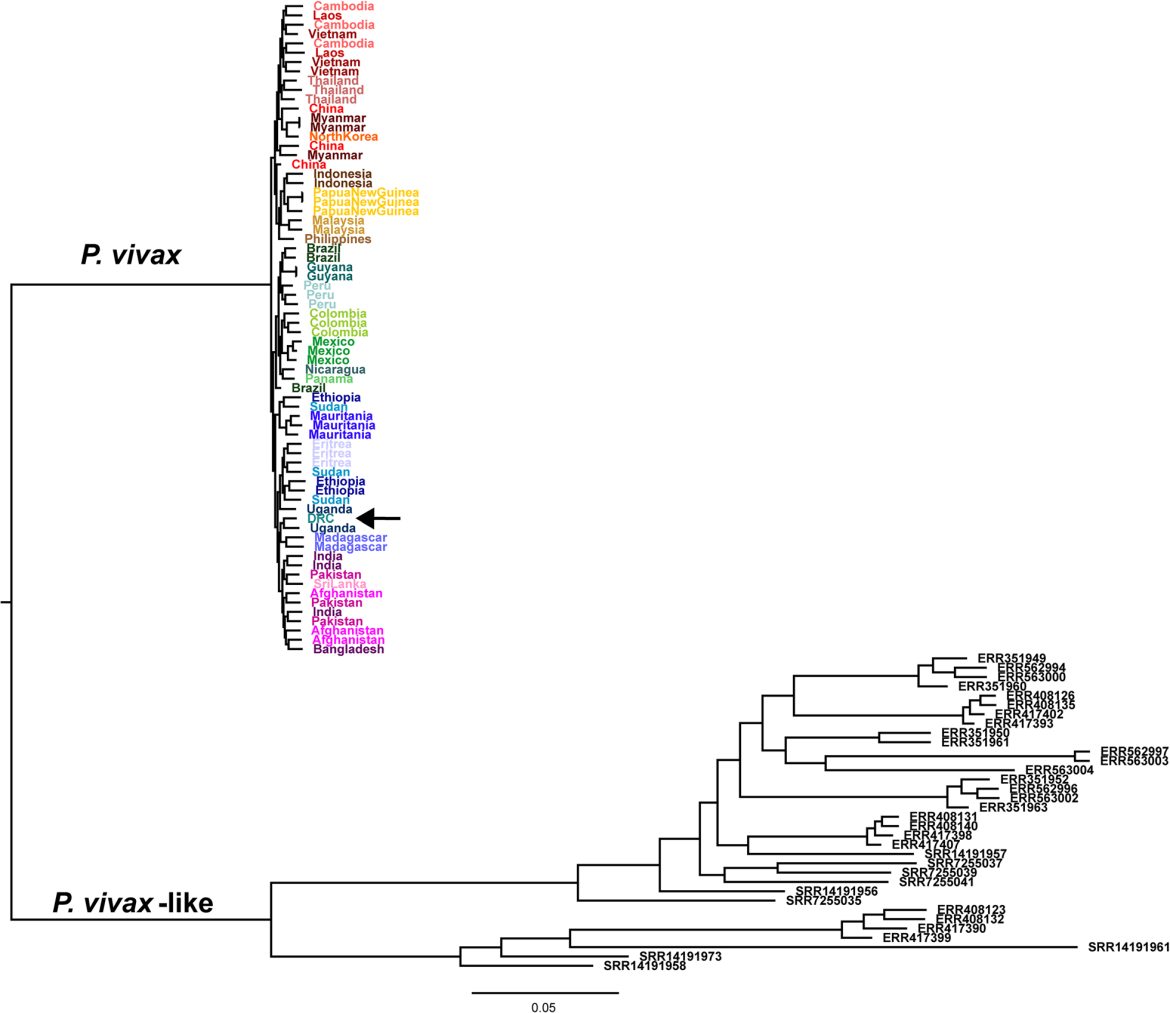
Maximum likelihood tree shows DRC *P. vivax* sample branches with *P. vivax* populations and not *P. vivax*-like. Maximum Likelihood tree of nuclear genome SNP data shows that *P. vivax* from the DRC does not branch with *P. vivax*-like samples (black tip labels). The DRC sample, indicated here with an arrow, clusters with other African samples

To assess the relatedness of *P. vivax* in humans in central Africa to the *P. vivax*-like malaria species found in non-human primates, a maximum likelihood tree was constructed including publicly available *P. vivax*-like genome sequences mapped to the PvP01 reference genome. Figure 2 shows that the *P. vivax* sample from the DRC clusters with other African *P. vivax* samples, while all *P. vivax*-like samples are separate from *P. vivax* populations. Additionally, the longer branch lengths for the *P. vivax*-like samples in Fig. 2 illustrate the higher level of diversity within this species than is found in any population of the human-infecting *P. vivax*. This suggests *P. vivax* has been separate from *P. vivax*-like for an extended period of time, and that the *P. vivax*-like populations are likely much older, much larger, or both older and larger than *P. vivax*.

### Copy number variation is present in binding proteins

**Fig. 3:**
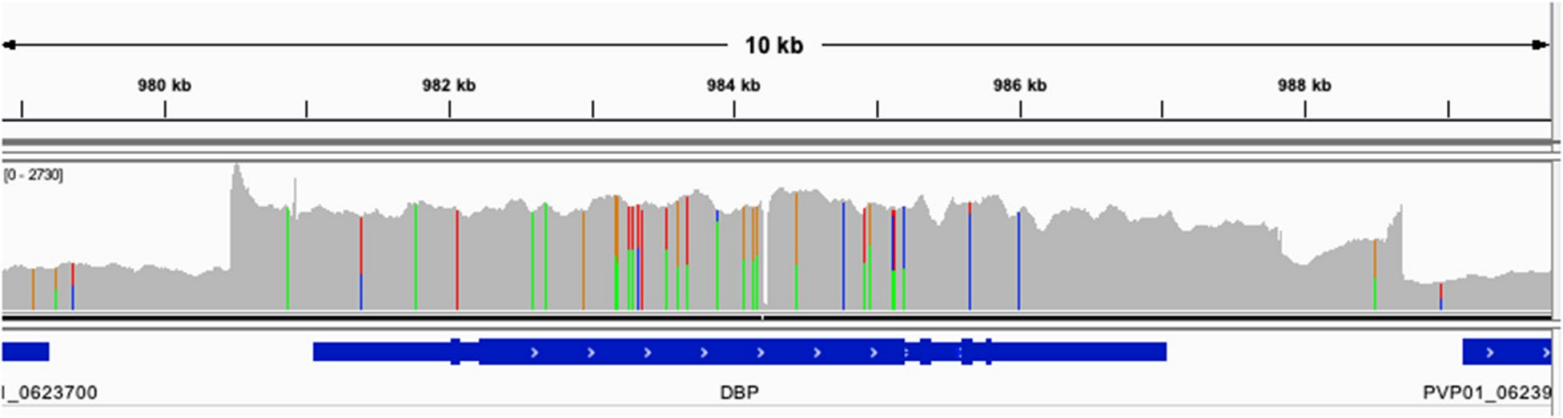
Read Pileup image of duplication of PvDBP in *P. vivax* from DRC. IGV view of the read depth for PvDBP in the DRC *P. vivax* sample where genome coordinate is on the X-axis and read depth is on the Y-axis. This shows an increase in read depth around PvDBP indicating a duplication, and the different variants present along this region suggest two distinct haplotypes

Copy Number Variation (CNV) in certain binding proteins is potentially important for pathogenesis of *P. vivax* in Duffy-negative individuals [15]. BAM files aligned to PvP01 with optical duplicates removed were used to compare read depth within the gene region to coverage in the region 10 Kb upstream and downstream of the coding region for several genes related to erythrocyte binding and invasion: PvDBP, PvDBP2, PvRBP1a, PvRBP1b, PvRBP2a, PvRBP2b, and PvRBP2c based on results from [15]. In the DRC *P. vivax* sample, only one gene, PvDBP, had evidence of a potential gene duplication (Additional file 1: Table S2). Lumpy was used to determine the number of paired-end and split reads that support a duplication in PvDBP, which showed evidence of a duplication of 8216 base pairs in length at chr6: 980,472–988,688 with 292 paired-end reads and 419 split reads supporting the structural variant. The ratio of the coverage for the duplicated PvDBP region compared to the surrounding intergenic region was 2.47. Based on the IGV pileup view in Fig. 3, there appears to be two distinct copies of this gene being mapped to the single PvDBP reference annotation. The region of higher read depth extends into the intergenic regions on either side of the gene annotation for PvDBP and is consistent with the duplication type first reported in Malagasy samples [48, 49].

## Discussion

Despite the publication of recent studies on *P. vivax* diversity that include African samples [33, 48, 50–52], there is still very little known about this pathogen in central Africa. Analyses of one new *P. vivax* genome collected from the Idjwi island of Lake Kivu in DRC show that this sample falls within the scope of African parasite diversity and is distinct from *P. vivax*-like samples. This suggests that an endemic *P. vivax* population is present in central Africa, as previously proposed by Brazeau et al*.* [6].

The results shown in Fig. 1 suggest the DRC *P. vivax* sample is most like those from Uganda and Madagascar. While the similarity between *P. vivax* from eastern DRC and Uganda is not surprising, it is interesting that *P. vivax* populations in the DRC, Uganda, and Madagascar all share ancestry with South Asian samples, as shown in Fig. 1C, D, but no measurable ancestry from Southeast Asian *P. vivax* populations despite a well-documented history of Austronesian human migration into this region [53–55].

The phylogenetic tree in Fig. 1B shows that this new *P. vivax* sample clusters with other African samples and not with any *P. vivax*-like samples that have been sequenced previously, suggesting that there is a *P. vivax* population in the DRC separate from potential zoonosis from an animal reservoir. However, all publicly available genome-wide *P. vivax*-like sequences to date have been collected from animals in countries on the West coast of Africa [13, 14]. The only *P. vivax*-like sample collected from a human infection was sequenced for two mitochondrial genes, which limits its utility compared to genome-wide sequencing assays [11]. Further sampling of both humans and non-human primates throughout the broad geography of central Africa is needed to determine whether there truly is no transfer of parasites across species. Though one population screen performed in Gabon found no evidence of cross-species infection of *P. vivax*-like in humans, in vitro studies indicate that there is little host specificity of *P. vivax*-like, suggesting Duffy-positive individuals living in this region may be susceptible to infection [13, 56].

These analyses replicate previous studies showing that global *P. vivax* populations are distinct from each other based on geographic distance, and most sharing of haplotype backgrounds occurs within geographic regions and only rarely across geographic borders. This geographic separation of ancestral groups, along with the summary statistics calculated in Additional file 1: Table S1 and illustrated in Additional file 1: Figs. S1 and S2, possibly indicate that DRC has a comparable *P. vivax* population size relative to Ethiopia and Uganda, though this interpretation is greatly limited by the reductive nature of genome-wide summary statistics.

Though the evidence linking copy number of Duffy binding ligand genes with *P. vivax* infection of Duffy-negative individuals is not conclusive [18, 19], it remains a subject of concern, especially since the Duffy Binding Protein-II domain is one of the foremost vaccine target candidates [20]. Results indicate this *P. vivax* sample from the DRC has a duplication in PvDBP relative to the PvP01 reference genome that corresponds with the longer Malagasy-type duplication, as opposed the shorter PvDBP duplication first detected in Cambodian samples [49]. Duplications in PvDBP may play a role in Duffy-independent mechanisms of infection in Duffy-negative individuals and should be considered in future studies [19].

These findings are largely limited by the uncertainty of whether this single sample is representative of the larger population of *P. vivax* in central Africa. This *P. vivax* sample was collected from an individual with no known travel history in a region with an estimated 98% homozygosity for Duffy-negativity [17], however as the patient’s Duffy genotype was not collected in the study, caution should be exercised until future studies can provide further context.

It has become clear from epidemiological studies that *P. vivax* is much more common in central Africa than previously thought [6, 9, 10, 52]. Generating genomes from these infections however is difficult, as most of them are extremely low parasite densities [6]. Thus, they are not amenable to whole genome sequencing. To increase understanding of this parasite in Africa, the research community needs to continue to try to identify samples amenable to analysis and deposit them for community use. Intentional sampling across Africa would further contextualize this sample within African *P. vivax* diversity and shed light on the mechanisms of infection in Duffy negative individuals.

### Supplementary Information

**Additional file 1: Figure S1.** *P. vivax* genome private alleles as a measure of population variation, separated by continent. **Figure S2.** Genome-wide Nucleotide Diversity within Africa. **Table S1.** *P. vivax* population diversity summary statistics, calculated across 1 Kb—long windows along the genome, excluding hyper-variable sites. Private alleles are the number of SNPs unique to that population; segregating sites are the sites that differ from PvP01 reference genome and which are not present at 100% frequency within the population. **Figure S3.** Admixture analysis results for all population sizes. **Table S2.** Identification of potential gene duplications in DRC *P. vivax* using read depth. **Figure S4.** Duplication of PvDBP in African samples. **Table S3.** PvDBP coverage for all African countries used to generate Fig. 3B. **Table S4.** F4 statistics calculated using Admixtools2. **Figure S5.** Phylogenetic tree labeled with both country and individual sample accession numbers. SH-aLRT and UFBoot support values generated by IQTree are shown on the node in the format: SH-aLRT support (%)/ultrafast bootstrap support (%). Nodes labeled with a dot and larger text correspond with the labelled nodes labeled in Fig. 1B.

**Additional file 2:** Sample accession numbers and location metadata of all Plasmodium vivax and Plasmodium vivax-like whole genome sequencing data used in this study.

## Data Availability

*Plasmodium vivax* Whole Genome Sequencing data from the Democratic Republic of the Congo available under BioProject accession: PRJNA909777. Accession numbers for previously published data used in this study are available in Additional file 2: Table S5 and on the project GitHub repository.
