# Supplementary material for: Genomic insights into Plasmodium vivax population structure and diversity in central Africa
Source: Malar J. 2024 Jan 18;23:27. doi: 10.1186/s12936-024-04852-y (PMC10797969; doi:10.1186/s12936-024-04852-y)
Supplement: Supplementary file 1 — Additional file 1: Figure S1. P. vivax genome private alleles as a measure of population variation, separated by continent. Figure S2. Genome-wide Nucleotide Diversity within Africa. Table S1. P. vivax population diversity summary statistics, calculated across 1 Kb—long windows along the genome, excluding hyper-variable sites. Private alleles are the number of SNPs unique to that population; segregating sites are the sites that differ from PvP01 reference genome and which are not present at 100% frequency within the population. Figure S3. Admixture analysis results for all population sizes. Table S2. Identification of potential gene duplications in DRC P. vivax using read depth. Figure S4. Duplication of PvDBP in African samples. Table S3. PvDBP coverage for all African countries used to generate Fig. 3B. Table S4. F4 statistics calculated using Admixtools2. Figure S5. Phylogenetic tree labeled with both country and individual sample accession numbers. SH-aLRT and UFBoot support values generated by IQTree are shown on the node in the format: SH-aLRT support (%)/ultrafast bootstrap support (%). Nodes labeled with a dot and larger text correspond with the labelled nodes labeled in Fig. 1B. [file 12936_2024_4852_MOESM1_ESM.docx]

**Additional file 1**

**
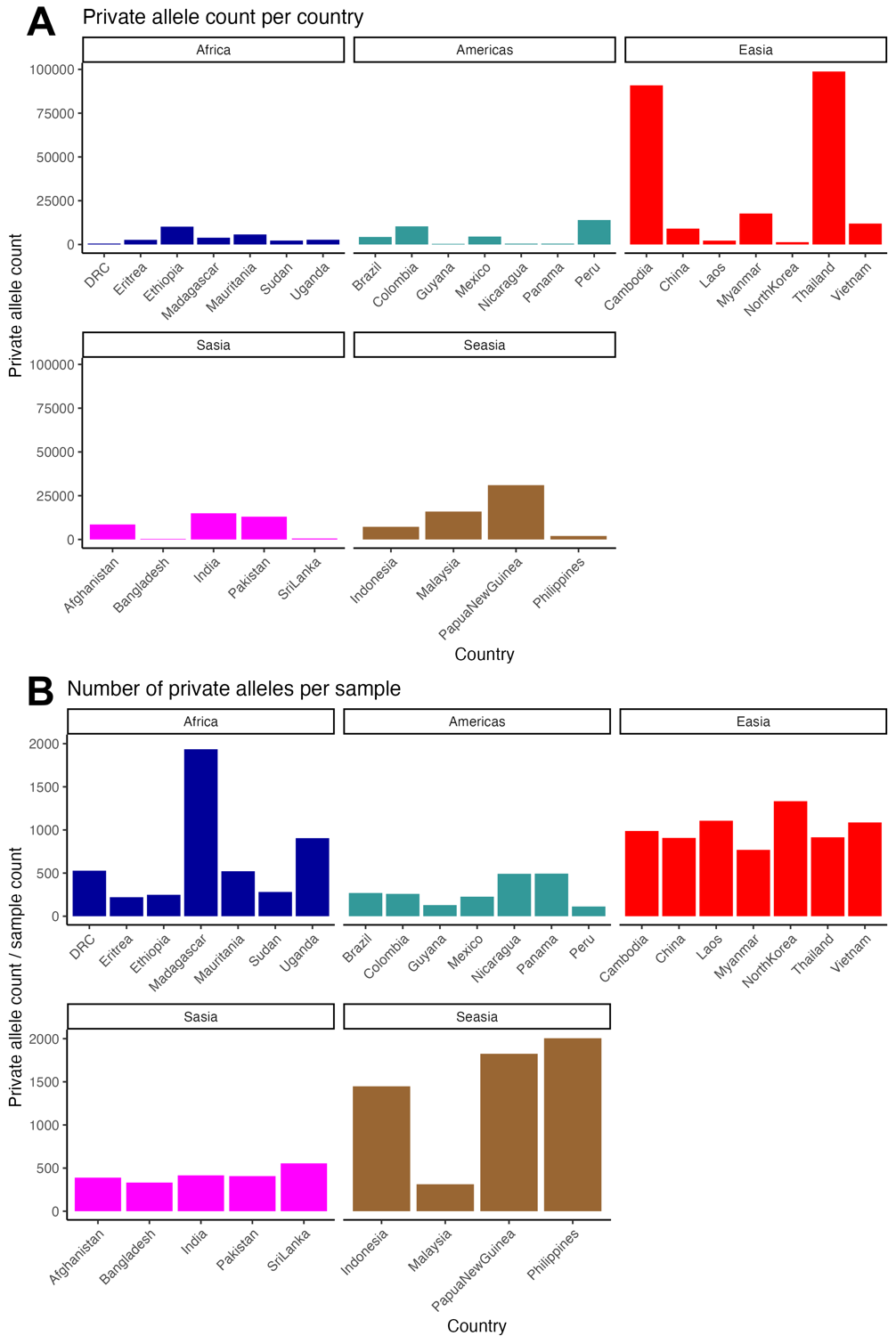
**

**Figure S1.** ***P. vivax* genome private alleles as a measure of population variation, separated by continent.**

**A.** Absolute count of private alleles for *P. vivax* in each country. *P. vivax* from the DRC has relatively few SNPs that are unique to this population (528 SNPs).

**B.** Private allele count is normalized by dividing by the number of samples in the population. This figure indicates that P. vivax in DRC has a similar private allele count to other African populations, except for Madagascar, once the count is adjusted for the number of samples.


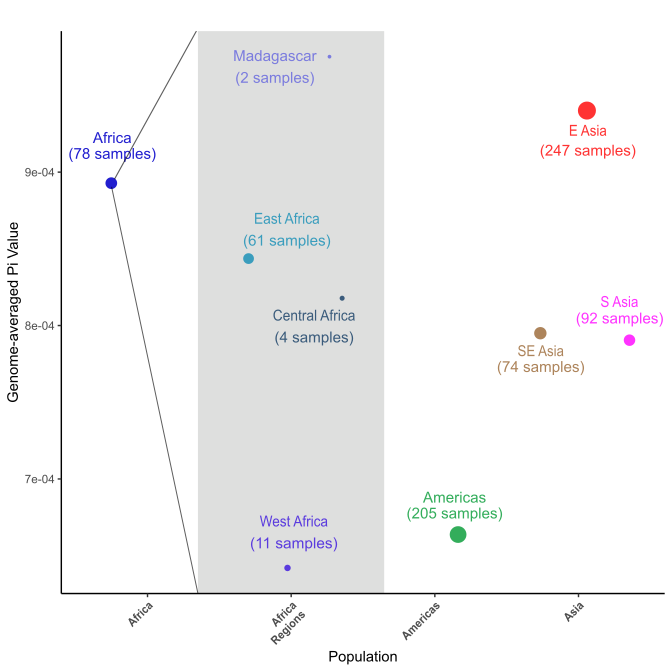


**Figure S2.** **Genome-wide Nucleotide Diversity within Africa**

Genome-wide average nucleotide diversity displayed by continent and by sub-region. African *P. vivax* samples grouped together have similar genome-wide diversity as populations in Asia. Within Africa, nucleotide diversity of central African samples (DRC and Uganda) is similar but slightly lower than that of East African (Ethiopia, Eritrea, and Sudan). (Pi values: Africa = 8.9267e-4; Americas = 6.6364e-4; East Asia = 9.4076e-4; South Asia = 7.9435 e-4; Southeast Asia = 7.9125e-4)

| **Region** | **Country** | **Number of Private Alleles** | **Sample Count** | **Segregating Sites** | **Private Alleles Per Sample** |
| --- | --- | --- | --- | --- | --- |
| Africa | Democratic Republic of Congo | 528 | 1 | 20755 | 528 |
| Africa | Eritrea | 2648 | 12 | 49750 | 220.666667 |
| Africa | Ethiopia | 10195 | 41 | 82966 | 248.658537 |
| Africa | Madagascar | 3871 | 2 | 18169 | 1935.5 |
| Africa | Mauritania | 5739 | 11 | 38829 | 521.727273 |
| Africa | Sudan | 2251 | 8 | 43634 | 281.375 |
| Africa | Uganda | 2715 | 3 | 23558 | 905 |
| Americas | Brazil | 4312 | 16 | 39158 | 269.5 |
| Americas | Colombia | 10362 | 40 | 61993 | 259.05 |
| Americas | Guyana | 388 | 3 | 294 | 129.333333 |
| Americas | Mexico | 4536 | 20 | 33466 | 226.8 |
| Americas | Nicaragua | 492 | 1 | 19040 | 492 |
| Americas | Panama | 494 | 1 | 18376 | 494 |
| Americas | Peru | 13974 | 124 | 66408 | 112.693548 |
| EAsia | Cambodia | 90890 | 92 | 215306 | 987.934783 |
| EAsia | China | 9077 | 10 | 57907 | 907.7 |
| EAsia | Laos | 2214 | 2 | 19180 | 1107 |
| EAsia | Myanmar | 17695 | 23 | 82611 | 769.347826 |
| EAsia | North Korea | 1333 | 1 | 19774 | 1333 |
| EAsia | Thailand | 98794 | 108 | 238360 | 914.759259 |
| EAsia | Vietnam | 11956 | 11 | 67515 | 1086.90909 |
| SAsia | Afghanistan | 8569 | 22 | 66479 | 389.5 |
| SAsia | Bangladesh | 332 | 1 | 20728 | 332 |
| SAsia | India | 14966 | 36 | 92994 | 415.722222 |
| SAsia | Pakistan | 13030 | 32 | 79154 | 407.1875 |
| SAsia | Sri Lanka | 555 | 1 | 22072 | 555 |
| SEAsia | Indonesia | 7239 | 5 | 38468 | 1447.8 |
| SEAsia | Malaysia | 15943 | 51 | 66571 | 312.607843 |
| SEAsia | Papua New Guinea | 31024 | 17 | 74267 | 1824.94118 |
| SEAsia | Philippines | 2005 | 1 | 14277 | 2005 |

**Table S1.** *P. vivax* population diversity summary statistics, calculated across 1Kb- long windows along the genome, excluding hyper-variable sites. Private alleles are the number of SNPs unique to that population; segregating sites are the sites that differ from PvP01 reference genome and which are not present at 100% frequency within the population.


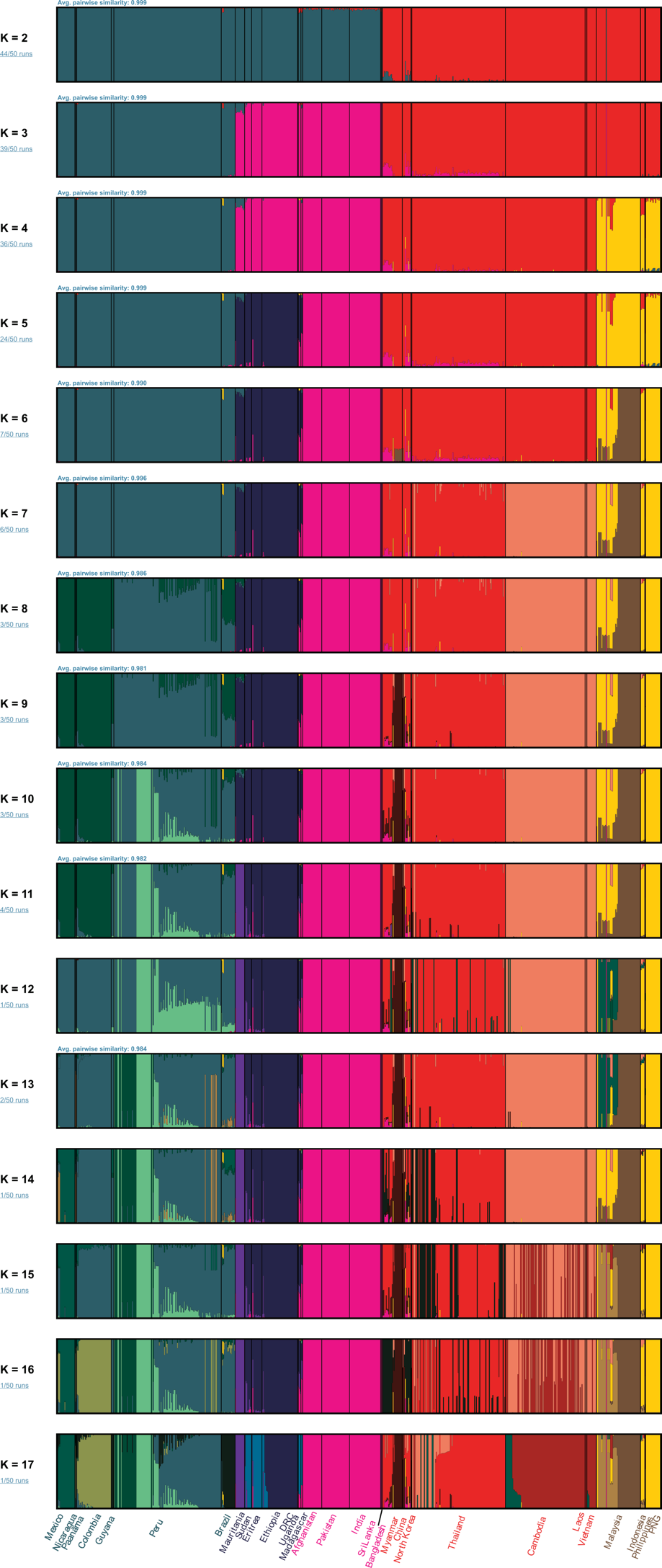


**Figure S3.** **Admixture analysis results for all population sizes**

Results of admixture analysis performed for putative population sizes of 2 through 17 (K=2 through K=17). Populations are primarily separated by geography, with little shared ancestry between regions.

| Gene | Chr | Upstream  Coordinates | Gene  Coordinates | Downstream  Coordinates | Gene  Read Depth | Non-Genic  Read Depth | Coverage  Ratio |
| --- | --- | --- | --- | --- | --- | --- | --- |
| PvDBP | LT635617 | 970025-980025 | 980025-988681 | 988681-998681 | 4378.26707 | 1772.59359 | 2.47 |
| PvDBP2 | LT635612 | 94013-104013 | 104013-107429 | 107429-117429 | 2120.53731 | 1800.19293 | 1.18 |
| PvRBP1a | LT635618 | 61106-71106 | 71106-80980 | 80980-90980 | 2397.57195 | 2437.86531 | 0.98 |
| PvRBP1b | LT635618 | 49612-59612 | 59612-70478 | 70478-80478 | 2467.78531 | 2256.64292 | 1.09 |
| PvRBP2a | LT635625 | 102686-112686 | 112686-121381 | 121381-131381 | 2260.37328 | 1917.22883 | 1.18 |
| PvRBP2b | LT635619 | 23312-33312 | 33312-43704 | 43704-53704 | 2305.30347 | 1922.30887 | 1.2 |
| PvRBP2c | LT635616 | 1448611-1458611 | 1458611-1467388 | 1467388-1477388 | 1271.57553 | 1645.0383 | 0.77 |

**Table S2. Identification of potential gene duplications in DRC *P. vivax* using read depth.** Read depth for each gene was determined for the DRC *P. vivax* sample using the BAM file with optical duplicates removed. Read depth for the non-genic (upstream and downstream) regions and within the gene itself was estimated by Bedtools, and the coverage ratio is the result of dividing the gene read depth by the non-genic read depth.


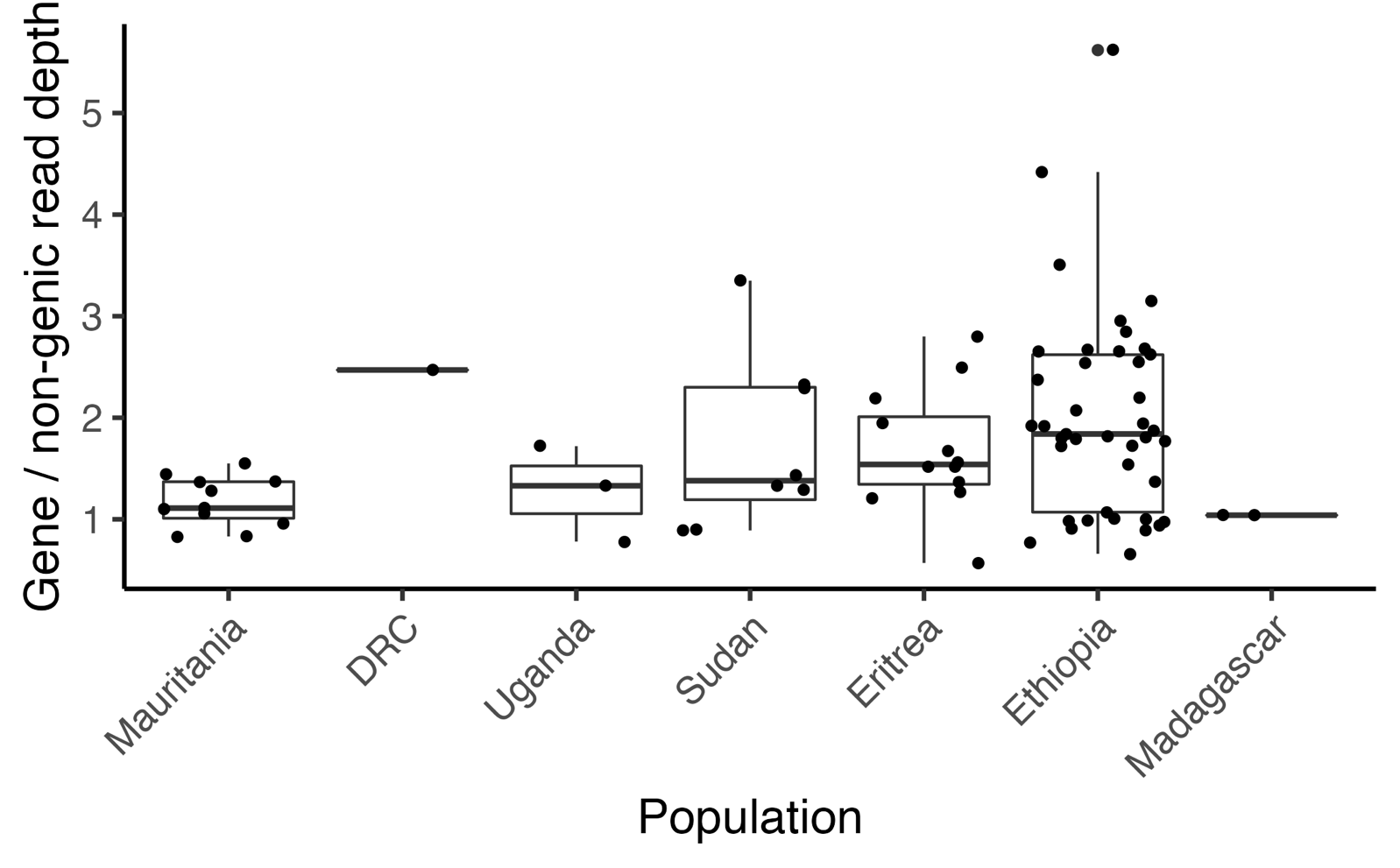


**Figure S4. Duplication of PvDBP in African samples**

PvDBP copy number variation for *P. vivax* in each country in Africa. Most countries have a mix of single copy and one or more duplications except for Mauritania and Madagascar.

| **Accession** | **Country** | **Genic**  **Coverage** | **Non-Genic**  **Coverage** | **Coverage**  **Ratio** |
| --- | --- | --- | --- | --- |
| SANRU | DRC | 4378.26707 | 1772.59359 | 2.47 |
| ERR5740704 | Eritrea | 2.14381425 | 1.68838116 | 1.27 |
| ERR5740712 | Eritrea | 7.65415271 | 5.01969803 | 1.52 |
| ERR5740715 | Eritrea | 4.46517269 | 1.59574043 | 2.8 |
| ERR5740716 | Eritrea | 5.67136421 | 3.39906009 | 1.67 |
| ERR5740721 | Eritrea | 0.5459166 | 0.96065394 | 0.57 |
| ERR5740723 | Eritrea | 7.35185399 | 2.94860514 | 2.49 |
| ERR5740726 | Eritrea | 5.63532402 | 3.61338866 | 1.56 |
| ERR5740729 | Eritrea | 5.66270071 | 3.7219778 | 1.52 |
| ERR5740740 | Eritrea | 2.76377498 | 1.26382362 | 2.19 |
| ERR5740742 | Eritrea | 3.27318933 | 1.68043196 | 1.95 |
| ERR5740743 | Eritrea | 2.9459397 | 2.14563544 | 1.37 |
| ERR5740851 | Eritrea | 1.12579416 | 0.92780722 | 1.21 |
| ERR2678989 | Ethiopia | 313.21035 | 70.8569143 | 4.42 |
| ERR2678994 | Ethiopia | 39.3205498 | 40.2654235 | 0.98 |
| ERR2678996 | Ethiopia | 199.716415 | 74.8611639 | 2.67 |
| ERR2678997 | Ethiopia | 14.5378307 | 7.5840416 | 1.92 |
| ERR2678998 | Ethiopia | 65.544877 | 67.9140586 | 0.97 |
| ERR2678999 | Ethiopia | 123.29479 | 68.5157984 | 1.8 |
| ERR2679000 | Ethiopia | 1096.21763 | 194.911109 | 5.62 |
| ERR2679001 | Ethiopia | 30.2270995 | 11.8430157 | 2.55 |
| ERR2679002 | Ethiopia | 306.558739 | 116.855165 | 2.62 |
| ERR2679003 | Ethiopia | 153.339841 | 57.7811719 | 2.65 |
| ERR2679004 | Ethiopia | 1553.42694 | 442.006749 | 3.51 |
| ERR2679005 | Ethiopia | 135.929883 | 135.295171 | 1 |
| ERR2679008 | Ethiopia | 102.424165 | 32.4782522 | 3.15 |
| ERR2679009 | Ethiopia | 98.6847638 | 37.2579242 | 2.65 |
| ERR2679012 | Ethiopia | 33.4857341 | 17.4821018 | 1.92 |
| ERR5740701 | Ethiopia | 2.13029918 | 0.96965304 | 2.2 |
| ERR5740702 | Ethiopia | 2.95749105 | 1.91580842 | 1.54 |
| ERR5740709 | Ethiopia | 1.55307843 | 1.71157884 | 0.91 |
| ERR5740710 | Ethiopia | 1.02968696 | 1.56709329 | 0.66 |
| ERR5740727 | Ethiopia | 9.57560356 | 4.04529547 | 2.37 |
| ERR775189 | Ethiopia | 50.1778907 | 24.2323768 | 2.07 |
| ERR775190 | Ethiopia | 24.7500289 | 13.5826917 | 1.82 |
| ERR775191 | Ethiopia | 123.571214 | 67.3077192 | 1.84 |
| ERR925409 | Ethiopia | 77.1175927 | 27.0638436 | 2.85 |
| ERR925411 | Ethiopia | 53.2547072 | 28.5268473 | 1.87 |
| ERR925412 | Ethiopia | 45.92688 | 25.3045196 | 1.81 |
| ERR925416 | Ethiopia | 18.8858727 | 17.7147285 | 1.07 |
| ERR925417 | Ethiopia | 42.360633 | 21.8155185 | 1.94 |
| ERR925421 | Ethiopia | 8.97031304 | 9.49540046 | 0.94 |
| ERR925424 | Ethiopia | 19.300104 | 10.9335566 | 1.77 |
| ERR925431 | Ethiopia | 45.0070463 | 26.1352365 | 1.72 |
| ERR925433 | Ethiopia | 88.5899272 | 34.8744626 | 2.54 |
| ERR925435 | Ethiopia | 36.4942821 | 21.1715329 | 1.72 |
| ERR925436 | Ethiopia | 35.6503408 | 19.9080092 | 1.79 |
| ERR925438 | Ethiopia | 103.39136 | 38.509899 | 2.68 |
| ERR925439 | Ethiopia | 72.5393323 | 24.5691931 | 2.95 |
| ERR925440 | Ethiopia | 27.5119556 | 27.8035197 | 0.99 |
| ERR925441 | Ethiopia | 88.8489084 | 88.2980702 | 1.01 |
| SRR14191981 | Ethiopia | 12.8861037 | 9.39046095 | 1.37 |
| SRR14191982 | Ethiopia | 15.5872704 | 20.3619138 | 0.77 |
| SRR14191983 | Ethiopia | 6.57179161 | 7.36591341 | 0.89 |
| ERR490350 | Madagascar | 134.642717 | 130.056644 | 1.04 |
| SRR570031 | Madagascar | 513.619152 | 495.763424 | 1.04 |
| SRR14191966 | Mauritania | 19.6048285 | 12.6476852 | 1.55 |
| SRR14191967 | Mauritania | 14.3873166 | 15.019948 | 0.96 |
| SRR14191970 | Mauritania | 35.0400832 | 24.3187681 | 1.44 |
| SRR14191972 | Mauritania | 10.5723692 | 12.7894211 | 0.83 |
| SRR14191975 | Mauritania | 31.9665011 | 23.3059694 | 1.37 |
| SRR14191976 | Mauritania | 10.4561626 | 9.3930107 | 1.11 |
| SRR14191977 | Mauritania | 20.7820261 | 19.5213479 | 1.06 |
| SRR14191978 | Mauritania | 8.51957953 | 10.3180682 | 0.83 |
| SRR14191979 | Mauritania | 39.9512533 | 31.3178182 | 1.28 |
| SRR14191980 | Mauritania | 34.549151 | 25.2717228 | 1.37 |
| SRR332413 | Mauritania | 520.312579 | 472.131287 | 1.1 |
| ERR5740714 | Sudan | 2.57560356 | 1.92985701 | 1.33 |
| ERR5740717 | Sudan | 4.09587617 | 4.55479452 | 0.9 |
| ERR5740718 | Sudan | 5.42093104 | 2.32651735 | 2.33 |
| ERR5740728 | Sudan | 10.1383851 | 3.02189781 | 3.35 |
| ERR5740852 | Sudan | 1.72115051 | 1.93705629 | 0.89 |
| SRR14191963 | Sudan | 11.8701629 | 9.21417858 | 1.29 |
| SRR14191964 | Sudan | 20.3321012 | 8.8739626 | 2.29 |
| SRR14191965 | Sudan | 17.4111124 | 12.1355864 | 1.43 |
| ERR5740703 | Uganda | 3.10511725 | 2.33686631 | 1.33 |
| ERR5740705 | Uganda | 0.97400947 | 1.25312469 | 0.78 |
| ERR5740706 | Uganda | 2.90666513 | 1.6850315 | 1.72 |

**Table S3. PvDBP coverage for all African countries used to generate figure 3B.** See Table S2 for PvDBP coordinates used.

| **pop1** | **pop2** | **pop3** | **pop4** | **est** | **se** | **z** | **p** |
| --- | --- | --- | --- | --- | --- | --- | --- |
| pvl | DRC | PNG | Uganda | 0.0031888 | 1.2772E-04 | 24.9661983 | 1.424E-137 |
| pvl | DRC | PNG | Ethiopia | 0.00280492 | 1.4810E-04 | 18.93982009 | 5.358E-80 |
| pvl | DRC | PNG | Madagascar | 0.00274388 | 1.1186E-04 | 24.53023864 | 7.030E-133 |
| pvl | DRC | PNG | Eritrea | 0.00257469 | 1.2403E-04 | 20.75865454 | 1.024E-95 |
| pvl | DRC | PNG | Pakistan | 0.00252949 | 1.2462E-04 | 20.29780614 | 1.345E-91 |
| pvl | DRC | PNG | Afghanistan | 0.00251222 | 1.2886E-04 | 19.49594722 | 1.188E-84 |
| pvl | DRC | PNG | SriLanka | 0.00249463 | 1.4271E-04 | 17.48048597 | 2.018E-68 |
| pvl | DRC | PNG | Bangladesh | 0.00249387 | 1.4986E-04 | 16.6413591 | 3.496E-62 |
| pvl | DRC | PNG | India | 0.00248823 | 1.2973E-04 | 19.18062832 | 5.373E-82 |
| pvl | DRC | PNG | Sudan | 0.00237983 | 1.3586E-04 | 17.51681328 | 1.066E-68 |
| pvl | DRC | PNG | Guyana | 9.85E-04 | 1.0713E-04 | 9.198170239 | 3.641E-20 |
| pvl | DRC | PNG | Brazil | 9.42E-04 | 9.2782E-05 | 10.1498476 | 3.319E-24 |
| pvl | DRC | PNG | Nicaragua | 9.41E-04 | 9.2708E-05 | 10.15543284 | 3.134E-24 |
| pvl | DRC | PNG | Colombia | 9.32E-04 | 1.0243E-04 | 9.101371202 | 8.920E-20 |
| pvl | DRC | PNG | Thailand | 9.27E-04 | 7.5619E-05 | 12.26356511 | 1.421E-34 |
| pvl | DRC | PNG | Mauritania | 9.00E-04 | 1.0978E-04 | 8.196586144 | 2.473E-16 |
| pvl | DRC | PNG | Peru | 8.84E-04 | 1.0074E-04 | 8.773900568 | 1.726E-18 |
| pvl | DRC | PNG | Panama | 8.26E-04 | 1.0662E-04 | 7.74650634 | 9.446E-15 |
| pvl | DRC | PNG | Myanmar | 8.07E-04 | 7.0737E-05 | 11.40500208 | 3.948E-30 |
| pvl | DRC | PNG | Mexico | 8.06E-04 | 1.0881E-04 | 7.404705339 | 1.314E-13 |
| pvl | DRC | PNG | China | 7.40E-04 | 7.3768E-05 | 10.03492804 | 1.070E-23 |
| pvl | DRC | PNG | Cambodia | 5.88E-04 | 6.4353E-05 | 9.133260076 | 6.647E-20 |
| pvl | DRC | PNG | Laos | 5.81E-04 | 9.3595E-05 | 6.212136475 | 5.227E-10 |
| pvl | DRC | PNG | Malaysia | 5.16E-04 | 7.5149E-05 | 6.869492877 | 6.443E-12 |
| pvl | DRC | PNG | Vietnam | 5.07E-04 | 6.4147E-05 | 7.906284601 | 2.652E-15 |
| pvl | DRC | PNG | Indonesia | 2.74E-04 | 3.7911E-05 | 7.223936941 | 5.050E-13 |
| pvl | DRC | PNG | Philippines | 2.22E-04 | 9.6136E-05 | 2.313679263 | 2.069E-02 |
| pvl | DRC | PNG | NorthKorea | 1.36E-04 | 8.5669E-05 | 1.589632817 | 1.119E-01 |

**Table S4. F4 statistics calculated using Admixtools2.**

**
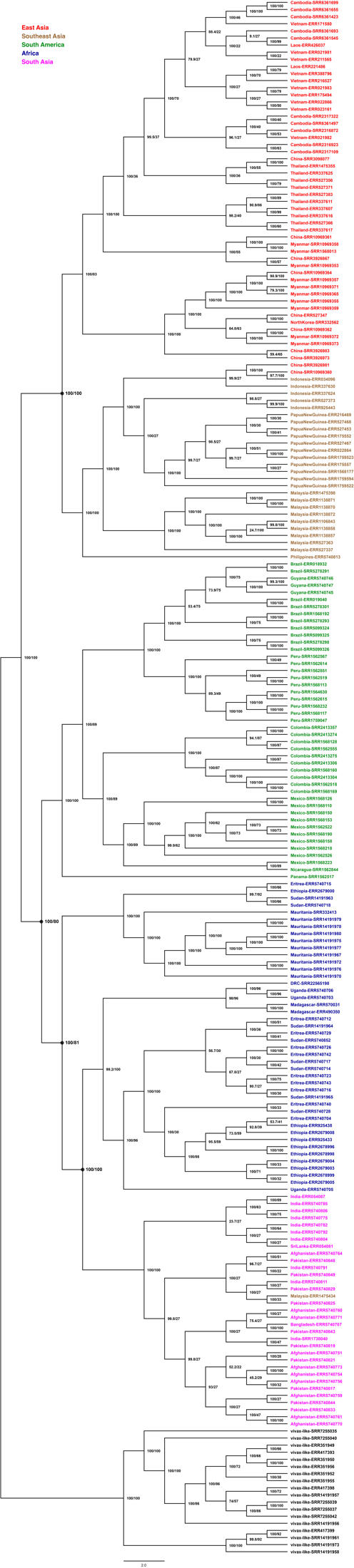
**

**Figure S5.** Phylogenetic tree labeled with both country and individual sample accession numbers. SH-aLRT and UFBoot support values generated by IQTree are shown on the node in the format: SH-aLRT support (%) / ultrafast bootstrap support (%). Nodes labeled with a dot and larger text correspond with the labelled nodes labeled in Figure 1B.
